# A Review of Serological Tests to Assist Diagnosis of Reactive Arthritis: Critical Appraisal on Methodologies

**DOI:** 10.3389/fimmu.2013.00418

**Published:** 2013-12-04

**Authors:** Tamara Tuuminen, Kari Lounamo, Marjatta Leirisalo-Repo

**Affiliations:** ^1^Department of Bacteriology and Immunology, Haartman Institute, University of Helsinki, Helsinki, Finland; ^2^Eastern Finland Laboratory Centre Joint Authority Enterprise (ISLAB), Mikkeli District Laboratory, Mikkeli, Finland; ^3^Department of Infectious Diseases, Health Centre of Lahti, Lahti, Finland; ^4^Institute of Clinical Medicine, University of Helsinki, Helsinki, Finland; ^5^Department of Medicine, Helsinki University Central Hospital, Helsinki, Finland

**Keywords:** serology, reactive arthritis, *Campylobacter*, *Salmonella*, *Yersinia*, *Chlamydia trachomatis*, cross-reactivity

## Abstract

On a population-based level, the incidence of reactive arthritis (ReA) is 0.6–27/100,000. The definition of ReA varies and its pathogenesis is not yet clear. Attempts in basic immunology to suggest hypotheses for proliferation of forbidden B cell clones, molecular mimicry, and involvement of cross-reactive antibodies are helpful but not sufficient. Importantly, for the clinical diagnosis of the preceding infection, serology is widely used. Unfortunately, the accuracy of associations between serologic findings and clinical conclusions is plagued by poor standardization of methods. So far, few attempts have been done to examine the pitfalls of different approaches. Here, we review several serologic techniques, their performance and limitations. We will focus on serology for *Yersinia, Campylobacter, Salmonella, Shigella*, and *Chlamydia trachomatis* because these bacteria have a longer history of being associated with ReA. We also address controversies regarding the role of serology for some other bacteria linked to autoimmune disorders.

## Introduction

Reactive arthritis (ReA) is a concept, not a well defined disease (1). It is an inflammatory sterile arthritis belonging to the group of arthritides known as the spondyloarthropathies which may develop within 2–4 weeks of the preceding gastrointestinal or genitourinary infections (1–3). The terminology, epidemiology, clinical presentation, antimicrobial treatment, and prognosis have been reviewed elsewhere (1, 3, 4). Evidence for infection triggering the arthropathy is most convincing when microbe isolation or antigen detection is successful. However, the search for the infection is often delayed and the microbe may no longer be culturable. Therefore, other options, e.g., serology are implied. Here, we will examine only methodological aspects of serology because, to our knowledge, such attempts have not yet been undertaken. No review of literature can be exhaustive; rather we will pick up examples of methodological solutions and demonstrate their variability.

## Serology for *Yersinia* Infections

The first mention of “reactive arthritis” was done by Finnish physicians in 1969 who described ReA as sterile arthritis after infection with *Yersinia enterocolitica* (5).

An indirect hemagglutination test (IHA) was among the first serological tests (6, 7) which is still in use albeit erythrocytes were substituted with particles. In IHA, erythrocytes are sensitized with heated extracts from bacteria. Antibodies to different *Y. enterocolitica* or *Y*. *pseudotuberculosi*s serotypes, or biotypes, or serogroups react with antigens and produce clumping. IHA and complement fixation (CF) detect primarily IgM-class antibodies because these are 10-valent compared to 2-valent IgG-class antibodies (having only two sites for specific antigen binding). As a rule of thumb, when the immune response is initiated, IgM-class antibodies reactive with LPS will appear first. The response is followed by a class switch and production of IgA- and IgG-class antibodies toward protein structures. Since both IgM and IgG may react in IHA or CF, these tests may not discriminate recent infection from past exposure unless IgM are inactivated by sample pre-treatment. Agglutination techniques are sensitive but their specificity could be suboptimal if a crude extract antigen is used. For instance, sera from brucellosis patients may react with particles sensitized with the *Y. enterocolitica* serotype O:9 antigen. Differentiation between these pathogens can be done, e.g., with EIA or immunoblot detecting antibodies against plasmid-encoded *Yersinia*-associated outer membrane proteins (OMPs) (8).

The result of a single agglutination technique may have a limited value. For example, in IHA human sera from blood donors contained antibodies to both *Yersinia* serotypes in titers 4-512 whereas patient samples produced antibody titers 512-2048 and 32-256 against *Y. enterocolitica* O:3 and *Y. enterocolitica* O:9, respectively (7). In Tanzania using microagglutination, *Y. enterocolitica* antibodies to the serotype O:3 were found in 2.6% of children and 0.9% of healthy adults, and to the serotype O:9 in 5.3 and 2.3%, respectively (9). But in countries where consumption of pork per capita is higher, seropositivity among healthy population could be also higher. Using EIA and immunoblot the seropositivity to *Yersinia* antigens was 19–31 and 33–43%, in Finnish and German healthy volunteers, respectively (10).

Using Immunoblot IgA antibodies to a 36-kDa protein were present in 18/19 ReA compared to 8/17 with non-arthritic yersiniosis. These antibodies persisted for 8–12 months (11). Although the difference is significant, these antibodies may not be a biomarker for the diagnosis of *Yersinia*-trigged ReA. Interestingly, the avidity maturation of IgA antibodies toward whole bacteria extracts or LPS was observed in ReA patients but not in healthy convalescents. This phenomenon was not demonstrated with antibodies toward plasmid-encoded proteins (12).

The interpretation of clinical significance of persisting IgG and IgA antibodies to different antigens remains an issue for scientific debate. The correlation of the persistence of *Yersinia* IgA to formalinized whole bacteria “OH” antigens (13, 14) with arthritis was not confirmed later when antibodies to plasmid-encoded antigens were studied in patients on the follow-up after 10 years (15). It was concluded that neither IgA nor IgG persistence have a discernable influence on the clinical course (15).

The matter of utmost interest is to know whether *Yersinia* truly persists. What defines “chronic *Y. enterocolitica* infection” (16, 17), and what are the symptoms of this clinical entity? If infection indeed persists in a latent form, why can’t we demonstrate its activation upon immunosuppression of any kind? Chronic infection was defined as a state of a negative culture and absence of agglutinins but the presence of IgA and IgG antibodies to 36 and 46 kDa virulence-associated proteins detected by immunoblot (16). By definition, antibody persistence is not the same as infection persistence; the question raised earlier (18). *Y. enterocolitica* O:3 was maintained for several weeks in primary cultures of human synovial cells or fibroblasts (19, 20), however these experimental models do not reflect the multi-functional, multi-cellular, and multi-organ model of the human body. Bacterial LPS, heat shock protein (HSP) (21) and the 16S ribosomal RNA sequences were detected in patients with *Y. enterocolitica* O:3 triggered ReA (17), however the proof of viable *Yersinia* in any body compartment is missing.

Today our knowledge on the pathogenesis of infections caused by *Yersinia* has been extended, and different adhesion molecules have been described (22). The most important are invasin (Inv), YadA (*Yersinia* adhesion A, previously known as Yop1), which is the major adhesin, and Ail. Besides, there are several other proteins, like YeuB, which also elicit immunological responses. After acute infection all classes of antibodies to YeuB, Ail, YadA, and Inv develop early with a peak on the second- third-weeks. These antibodies were more prevalent in patients with gastroenteritis compared to ReA patients (23). Yad of *Y. enterocolitica* has a collagen binding activity. By contrast, Yad of *Y. pseudotuberculosis* binds predominantly to fibronectin (22). Both species have been implicated in ReA, although, only *Y pseudotuberculosis* serotypes O:1a and O:3 have been reported to trigger ReA (3).

## Serology for *Campylobacter* Infections

Host-pathogen interaction has been recently reviewed (24, 25). *Campylobacter* serology is highly variable. For example, in 40 diarrheal patients in whom *C. jejuni* was isolated, the seropositivity was observed in 82, 62, and only 38% of patients, respectively when immunofluorescence, CF, and agglutination techniques were applied. Using paired sera samples from 15 patients a fourfold or greater rise of titers was observed in only five (26). In a comparison study between the CF and Western blot (WB) the seropositivity with CF was 88.6 and 28.5% for infected patients and blood donors, respectively. Seven immunoreactive antigens (range 14–67 kDa) were found, of which (29, 37, and 43 kDa) were detected by 86, 84, and 91% of infected patients, respectively (27).

In the early 1980s, EIA-based techniques emerged (28–30). One EIA (28) used heated and sonicated crude antigen preparations from six *C. jejuni* strains; the other (29) used a single *C. jejuni* isolate. In the latter, the common antigen was prepared by acid extraction which was cross-reactive between different *C. jejuni* and *C. coli* strains. In another EIA the acid extract was prepared from three strains (30). The acid extract assays have been used for routine diagnostics of *Campylobacter* infections ever since. Two immunodominant antigens were characterized in the acid extract as 30 and 60 kDa (30), however it could be assumed that it is a mixture of multiple water soluble proteins. These EIAs were evaluated with human sera (30, 31) and with sera from immunized rabbits (29). IgM and IgG-class antibodies were positive in only 73 and 52%, respectively in culture confirmed hospitalized patients (31). The seroprevalence to this antigen in healthy blood donors increases with age, reaching 60, 42, and 21% for the age group of 56–65 years for IgG, IgA, and IgM, respectively (32). These findings have an important implication for interpretation of a single positive result. Firstly, it is evident that, IgM-class antibodies may persist, making an assumption of acute infection on the basis of a single serum specimen imprecise. Secondly, diagnostic serology is more informative in younger subjects.

Also in the 1980s, an EIA based on a mixture of LPS (more precisely lipo-oligosaccharides (LOS)) from two strains was published (33). This method resulted in only 70% sensitivity when paired human sera were tested. When formalinized whole bacteria were used, IgG and IgM antibodies from patients with recent *C. jejuni*/*coli* enteritis were detected in 82 and 77%, respectively, while antibodies were detected in 5% of healthy blood donors. Sera from small children showed low reactivities. Interestingly, in a comparison study better discriminatory power was achieved with sonicated whole cell and ultracentrifuged sonicate than from acid glycine extract (34).

Later, an EIA to detect antibodies to a heat stable antigen (presumably LOS) was described (35). The authors analyzed more than 600 sera samples from 210 patients with recent infections and found that IgG, IgA, and IgM EIA sensitivities were 71, 60, and 80%, respectively at a specificity level of 90%. It was found that IgG-class antibodies persisted at least 4.5 months with great inter-individual variation, but for IgM and IgA antibodies decay of immune response was evident by 2 months from the onset of the infection.

Recently, a number of immunogenic antigens in *Campylobacter* have been described (24). The major immunodominant antigen is flagellin, the subunit protein of flagella. Also, major outer membrane proteins (MOMP), periplasmic-associated membrane proteins PEB1 (28 kDa), and PEB3 (30 kDa) as well as 47 and 84 kDa proteins are immunogenic. Novel methods detect antibodies to several recombinant proteins of *Campylobacter*, e.g., MOMP, PEB4, PEB2, PEB1 OMP18, P39 in a line blot format or conventional ELISA. For example, in pediatric patients with GBS serodiagnosis based on recombinant antigens yielded better specificity than tests using thermostable and whole cell-antigens (36). The same has not yet been shown for ReA.

Interestingly, *Campylobacter* infection can cause sequelae of the nervous system, i.e., Guillain–Barré (GBS)/Miller Fisher syndrome or lead to ReA. It has been reported that in GBS the antibodies to nerve GM1b gangliosides may be cross-reactive with the LOS of *Campylobacter* (24, 37). This may imply that *Campylobacter* serology for GBS may need different antigen preparations compared methods for ReA.

## Serology for *Salmonella* Infections

*Salmonella* can more likely than *Campylobacter* cause ReA (1, 3, 25, 38). The first serodiagnostic test for *Salmonella* was developed in 1896 after the observation that the sera of typhoid patients clumped formalin-fixed bacteria from the host (39). These bacterial preparations preserve the O-antigen and the Flagella. The test has undergone several refinements (40). The history and evaluation of this test has been recently reviewed (41). In the original test, sera from those who presented with fever were mixed with a suspension of killed *S. typhi* bacteria. If the antibodies (agglutinins) to bacterial structures were present, agglutination of the cell suspension occurred. Today, this test is used to support diagnosis of ReA. The test can demonstrate antibodies to O-, H-, or Vi-antigens separately. As a rule of thumb, anti-O IgM antibodies appear first, anti-H IgG antibodies are produced later and may persist, anti-Vi antibodies can detect carriers (42). The slide test modifies the Widal test, when the serum and cell suspension are mixed and clumps are scored. A more advanced modification is performed in tubes or microwells and requires an incubation step. The Widal test can produce false reactions due to variability in antigen preparation, too early sampling or technical difficulties of interpretation and it has poor standardization and reproducibility (41–43). False positive reactions might be common due to intrinsic cross-reactivity with malaria and *Enterobacteria* infections. Interestingly, in Nigerian patients who had a positive malaria smear, were unvaccinated against *S. typhi* and who had negative *S. typhi* stool isolation, the Widal test was positive in titers 1:40, 1:80, and 1:160 in 85, 12, and 3%, respectively. The corresponding figures in patients without malaria were 45, 15, and 10% (43). These data caution against the use of a single result to interpret *Salmonella* serology. As a matter of fact, historically, all tests based on the measurement of antibody titers have relied on the comparison of acute and convalescent sera. It has been emphasized that neither of the tests are interpretable unless the sensitivity and specificity of the test for the specific laboratory are known (43). The Widal test has lost some popularity in recent years (42). Disappointingly, also newer tests for IgM and IgG antibody detection lack sufficient sensitivity (max. 70%), and specify (max. 88%) when tested on samples of patients with confirmed Typhoid fever (44).

IHA utilizes erythrocytes sensitized with the *Salmonella* O-antigen. The sensitivity is only 62% and specificity 98.2% with positive and negative predictive values of 66.7 and 96.7%, respectively (42). Counter current immunoelectrophoresis is based on the visualization of a precipitation band of antigen-antibody complexes. This method has a sensitivity close to Widal test (42).

A new approach to study *Salmonella* serology has been developed (41). Extracted LPS and flagellar antigens from four different strains each were applied to the gels for SDS-PAGE and immunoblot. The reacting antibodies were detected by polyvalent anti-human antibody. This method allows visualization of bands against LPS and flagellin on separate gels, and the combination of bands allows conclusion not only on the presence of specific antibodies but to estimate the causative agent. The authors found that all patients with culture confirmed salmonellosis produced anti-LPS antibodies. Conversely, humoral response to flagellar “d” antigens was seen in only 67% of patients (41).

Demonstration of antibodies to LPS of salmonellae other than *S. typhi* is common practice, especially in *S. typhimurium* or *S. enteriditis* infections, the two predominant serogroups responsible for the majority of gastroenteritis in Europe. In patients who had salmonellosis the LPS antibodies were equally detected with antigens prepared from phenolic or trichloro acid extraction; however, in the control group these antibodies were also highly prevalent (45). Disappointingly, in veterinary studies using 937 samples and a mix-ELISA test, no significant association was found between positive serology and bacteriology (46).

## Serology for *Shigella* Infections

*Shigella flexneri, sonnei*, and *dysenteriae* were associated with ReA on the basis of positive culture in patients with diarrhea (1, 47). *Shigella* spp and *E. coli* are closely related bacteria that are not distinguished with MALDI TOF nor when sequencing short DNA fragments. Theoretically, antigen preparations as whole bacteria sonicate, crude lysate, or even fractions of outer membrane are not good enough for serology because antibodies may produce cross-reactivity to gut microbiota (48). Interestingly, arthritogenicity of *Shigella flexneri 2a* was not associated with the presence or absence of antibodies toward epitopes of the cell wall (49). Advanced Lumixex™ technology allowed simultaneous detection of specific antibodies to recombinant invasin plasmid antigens Ipa B, C, and D as wells as to LPS from *Shigella sonnei, flexneri 2a*, and *dysenteriae* (50). Although promising, this technology has not yet been widely evaluated to support ReA diagnosis.

## Serology for *Chlamydia trachomatis*

The causative role of *C. trachomatis* in ReA is universally accepted, although the pathogenesis is still unclear (51–54). It is assumed that genital infection with *C. trachomatis* can result in a long-term persistence of metabolically active organisms residing in synovial tissues (52, 54). Amazingly, however, genetic material of only ocular serovars of *C. trachomatis* has been found so far in synovial biopsies from arthritis patients (52). If true bacterial persistence is the core feature of the *C. trachomatis*-trigged arthritis, could this clinical entity be indeed referred as ReA? Or should it be regarded as a separate entity because by definition ReA is the reaction of the immunological system toward encountered (and probably cleared?) infection.

Microimmunofluorescence (MIF) to detect antibodies to *C. trachomatis* and *C. pneumoniae* was among the first serologic methods (55, 56). A huge body of seroepidemiological studies on *Chlamydia* using in-house and commercial MIF has been published. The method utilizes elementary bodies (EB) that are attached to the objective glass, to where specific immunoglobulins bind, and further detected. To detect IgG and IgA antibodies, EBs should be treated to remove LOS because they react with protein structures mostly. By contrast, IgM detection requires antigen with LOS because IgM to carbohydrates as an early immune response appear first. The advantage of MIF is its ability to discriminate immunoresponses to different serovars (57). In a multicenter study the interpretation of the results by MIF was found to be subjective, and highly variable (58).

In the 1990s EIA-based techniques emerged. Those employed treated EBs (59), or recombinant LOS antigens to detect genus-specific antibodies (60) or synthetic peptides from immunodominant MOMPs (61). Peptide-based immunoassay in diagnosis of *C. trachomatis* triggered ReA was found useful (62), however, the evidence of specific antibodies does not prove casualty. Some commercial EIAs utilize MOMP, or HSP 60 (cHSP60). New formats, e.g., line blot have also become available. Among five EIAs the MOMP-based methods were superior in studies on tubal infertility and extra uterine pregnancy studies (63) but the problems of standardization still remain unsolved. In our and other’s opinion (64) whatever serology for *C. trachomatis* is used, it is of a limited value for ReA.

## Questions to be Resolved

Some bacterial species of the gut or skin microbiota have been implicated as autoimmune triggers (65–70). The authors suggested that sub-clinical urinary tract infection (UTI) by virtue of molecular mimicry of the hemolysins of *P. mirabilis* and the amino acid motif of the HLA-B27 antigen (expressed also on chondrocytes) may perpetuate immunopathology through cross-reactive antibodies. The same argument for molecular mimicry has been proposed as a causative link between *Klebsiella pneumoniae* and ankylosing spondylitis (AS) in HLA-B27 positive subjects (71). The analysis of the literature raises more questions than provides answers, for example:

1. Why phylogenically non-related obligate or facultative intra-cellular *Salmonella, Campylobacter*, or *C. trachomatis* may trigger similar post-infectious arthropathies collectively called ReA? What do they have in common?
2. Why are closely related enterobacteria, i.e., *Proteus mirabilis* and *K. pneumoniae*, associated with different clinical entities, the first with RA and the latter with AS?
3. Why is *Salmonella* but not *E. coli* [with an exception of only a few case reports (72)], associated with ReA? And why *P. mirabilis* with UTI but not *E. coli*? Indeed, why has *E. coli* UTI been so rarely implicated in perpetuation of ReA, considering its extremely high incidence?
4. Why do urogenital and enteric gram-negative bacteria trigger ReA but many respiratory gram-negative don’t?
5. Why non-pathogenic ubiquitous *yersiniae* can not be “blamed” for association with ReA, or could they?
6. Why diarrheagenic strains of *E. coli* are implicated in ReA (3) but frequently encountered urinary *E. coli* are not?

## Concluding Remarks

Here, some controversies of conventional serology to support the diagnosis of ReA are illustrated. We advocate new studies to examine disease associations using microarray or proteomic platforms to characterize immune profiles in ReA patients.

## Conflict of Interest Statement

The authors declare that the research was conducted in the absence of any commercial or financial relationships that could be construed as a potential conflict of interest.
